# ‘Dignity and respect’: An example of service user leadership and co‐production in mental health research

**DOI:** 10.1111/hex.12963

**Published:** 2019-09-26

**Authors:** Alison Faulkner, Sarah Carr, Dorothy Gould, Christine Khisa, Trish Hafford‐Letchfield, Rachel Cohen, Claudia Megele, Jessica Holley

**Affiliations:** ^1^ Independent Service User/Survivor Researcher London UK; ^2^ Department of Mental Health, Social Work and Integrative Medicine Middlesex University London London UK; ^3^ Service for Quality Assurance and PSW Wiltshire Council London UK

**Keywords:** adult safeguarding, co‐production, mental health research, mental health service users, survivor research, user‐led research

## Abstract

This paper explores the methodological aspects of a user‐led study investigating mental health service user experiences of targeted violence and abuse (often called 'hate crime'). 'Keeping Control' was a 16‐month qualitative study, undertaken in the context of adult safeguarding reforms in England. By collecting data on service user concepts and experiences, the research sought to address a gap in research and practice knowledge relating to targeted violence, abuse and hostility against people with mental health problems. In this paper, we discuss the significance of the design and methodology used for this study, with a particular focus on the interviews with service users. The research was both user‐led and carried out in collaboration with practitioners and academics, a form of research co‐production. Our aim is to inform researchers, practitioners and policymakers about the value of user leadership in co‐productive research with practitioners, particularly for a highly sensitive and potentially distressing topic.

## 1 INTRODUCTION

This paper explores the methodological aspects of a user‐led study investigating service user experiences of targeted violence and abuse (often called 'hate crime').^1^ 'Keeping Control' was a 16‐month qualitative study, undertaken in the context of adult safeguarding reforms in England. The study was partly undertaken to inform the implementation of ‘The Care Act 2014: Statutory Guidance on Making Safeguarding Personal’ England.^2^ ^[updated]^ These new policy approaches to adult safeguarding under the Care Act 2014 determine that safeguarding is 'everybody's business' and that it should become more outcome‐focused and person‐centred.^3^ By collecting data on service user views and experiences, the research sought to address a gap in research and practice knowledge relating to targeted violence, abuse and hostility against people with mental health problems.

In this paper, we discuss the significance of the design and methodology used for this study, with a particular focus on the interviews with service users, informed by reflections from both participants and researchers. The research was both user‐led and carried out in collaboration with practitioner academics and survivor researchers in a form of co‐production. Our aim with this paper was to inform researchers, practitioners and policymakers about the value of service user leadership in co‐productive research, particularly for a highly sensitive and potentially distressing topic. The intention was to open up both real and virtual spaces for dialogue to take place between service users and the researchers, practitioners and policymakers who might be able to effect change in relation to adult safeguarding. The findings from the overall study, the practitioner findings and the UK literature scoping review are published elsewhere.^1^, ^4^

## 2 BACKGROUND TO THE STUDY

It is well documented that disabled people are at higher risk of experiencing ‘hate crime’ based on their disability, or ‘targeted violence and hostility’.^6^ ^1^ The literature scoping review for this study suggested that the situation for people who experience mental distress and targeted violence and abuse in England is complex and poorly understood.^4^ There are significant gaps in the research evidence concerning mental health service users’ perceptions and experiences of risk and safeguarding,^7^ and personal experiences of victimization and abuse. Mitchell and Glendinning^8^ suggest that this could reflect ‘the state's role and pre‐occupation with risk management’ rather than with seeking to understand service users’ perspectives and experiences of safety and risk.

The majority of research on adult safeguarding has explored practitioner concepts, systemic issues, service configuration and models of decision making.^9^, ^10^, ^11^, ^12^ It suggests that reactive and technical approaches to risk management and safeguarding are inadequate for person‐centred practice.^13^ Risk and safety are most commonly defined by practitioners and articulated using managerial language.^7^, ^8^, ^14^, ^15^

Evidence is beginning to show that people who experience mental distress may not feel that adult safeguarding or the legal protections relating to ‘hate crime’ apply to them.^16^ In addition, some findings suggest that advice on prevention and protection amounts to ignoring abuse or avoiding situations where violence, hostility or abuse may occur, thus potentially increasing social isolation.^6^, ^17^ The negative effects of failed help‐seeking can be detrimental to mental health.^4^ Risk‐averse cultures in mental health services can be disempowering for service users who have not been meaningfully involved in the processes of risk assessment, management and decision‐making processes that affect them.^18^, ^19^, ^20^, ^21^, ^22^

A study funded by the Joseph Rowntree Foundation^18^ identified that perceptions of risk and rights are significantly different for mental health service users compared to other disabled people and service users. They are more often themselves perceived as a source of risk, rather than being considered potentially 'at risk' in vulnerable situations. The study highlighted the need for more co‐production, service user involvement and user‐led approaches 'as ways for ensuring that the vision and views of service users are encapsulated in any policy or service and the delivery, monitoring and evaluation of that service'.^18^ ^p.295^

In this study, we explored how mental health service users experience and conceptualize targeted violence and abuse, safety, prevention and protection in relation to adult safeguarding. We were also aware of the need to understand people's help‐seeking behaviour and prevention strategies in order to inform practice. We felt the literature powerfully indicated the need for user‐led research and co‐production methods to address some of the identified gaps in knowledge. The aim of this approach was to enable service users to find the voice and the freedom with which to talk about these profoundly sensitive issues, and enable us to reach practitioners and policymakers with a view to effecting change.

## 3 THEORETICAL AND METHODOLOGICAL APPROACH TO THE STUDY

### 3.1 User‐led research and experiential knowledge

User‐led research refers to research that is led by people who use services, in this case mental health services. It can be distinguished from *user‐controlled* or survivor research through, for example, being based within non–user‐led organizations or institutions, where the budget and full control are not held by service users.^23^ Nevertheless, the term ‘user‐led’ is often used interchangeably with 'survivor research' and defined in relation to concepts of empowerment, equality and change:

[survivor research] is committed to challenging the disempowerment of mental health service users/survivors and supporting them to have a greater say in their lives and influence in the world in which they live.^24^ ^p.18^

Sweeney^25^ highlights the roots of survivor research within the mental health service user/survivor movement, the role of empowerment and the ethics and values underpinning survivor research developed over the last decade or so by service user and survivor researchers.^26^ Russo^30^ defines survivor research by the central role taken by experiential knowledge throughout the research process, from design to analysis and interpretation of findings.

The purpose of survivor research is to enable silenced voices to speak from the margins and have the space to be heard.^31^ As pointed out by Wallcraft,^32^ mental health service users have 'traditionally been excluded from creating the knowledge that is used to treat us, and many of us have suffered from the misunderstanding of our needs by people who have been taught to see us as by definition incapable of rational thought'.^32^ ^p.133^ So, in other words, it is not just about being heard, it is about becoming believed as 'credible knowers'.^33^ It was a pivotal part of this study to privilege experiential knowledge through the methods adopted.

Experiential knowledge has a significant contribution to make where some of the basic premises of professional knowledge are strongly contested, as in mental health where the biomedical model is widely disputed.^34^, ^35^ Beresford^36^ and Russo^30^ challenge the assumption underlying positivist research that the *greater* the distance from the experience under investigation, the more reliable the view: the 'shorter the distance there is between direct experience and its interpretation… the less distorted, inaccurate and damaging resulting knowledge is likely to be'.^36^ ^p.7^ Many authors argue for a fundamental paradigm shift in knowledge production towards valuing and legitimizing experiential knowledge.^21^, ^37^, ^38^, ^39^, ^40^

### 3.2 Co‐production

Co‐production is a relatively recent concept, more often associated with health and social care practice than research.^41^, ^42^, ^43^ Advocates of co‐production have a vision that incorporates the transformation of power relations, in a similar way to survivor or emancipatory research.^44^ The Social Care Institute for Excellence^45^ emphasizes the core of co‐production as being an equal partnership between service users and carers with professionals working towards shared goals.

For INVOLVE, co‐production means researchers, practitioners and 'members of the public' working together, sharing power and responsibility from the start to the end of the project, including the generation of knowledge.^46^ However, co‐produced research generally remains in the leadership of academic researchers. Our understanding of a co‐productive approach to research involves collaboration between service users and practitioner and academic allies to transform the potential of research to achieve meaningful change. In this project, the principal investigator was a service user researcher and the study was co‐produced with practitioner academics and survivor researchers; hence our use of both terms 'user‐led' and 'co‐produced'.

### 3.3 A note on language

Early on in the project, we discussed the language and terminology we preferred to use for mental health and distress. We established a shared belief in the importance of social understandings and approaches; hence, we adopted the terminology of 'mental distress' rather than mental illness or mental health problems. We occasionally use the term 'mental health problems' in this paper where the term has been used by other authors.

## 4 AIMS OF THE MAIN STUDY

The main study to which this paper relates aimed to explore:

- Mental health service user concepts and experiences of targeted violence and abuse (‘disability hate crime’), prevention and protection;
- Where mental health service users go to get support if they are frightened, threatened or have been victims of targeted violence and abuse;
- The responses of adult safeguarding, mental health and other relevant stakeholders to mental health service user concepts and experiences of targeted violence and abuse (‘disability hate crime’).

The main study findings are intended to help relevant practitioners and agencies to understand the role that targeted violence and abuse plays in the lives of mental health service users, their help‐seeking and prevention behaviour, and to place this knowledge at the centre of future practice. The main study findings are reported in Ref. 1 and the literature scoping review in Ref.[4]. The practitioner findings are reported in Ref.^5^.

### 4.1 Aims of this paper

**Figure 1:**
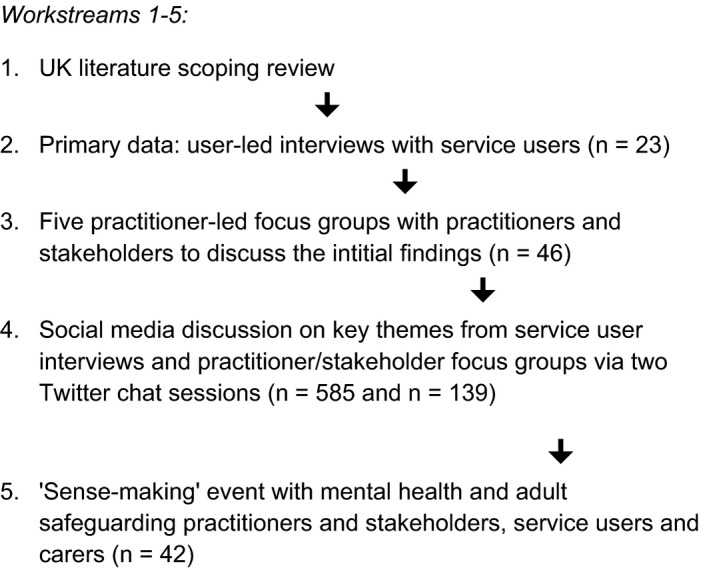
Study design structure

In this paper, we aim to describe the methods used and the process with which we conducted this study, with a focus on Workstream 2 (see Figure 1). We aim to demonstrate the value of adopting this approach in investigating a powerfully sensitive subject as it affects the lives of mental health service users. We incorporate the results of two approaches to reflective practice: a brief survey of participants' experiences and the shared reflections of the research team. In doing so, we are responding to the plea to improve the quality of how this kind of research is reported in order to gain a better insight into the methods and impact of involving service users in research.^47^

## 5 THE DESIGN OF THE MAIN STUDY

The study was designed to create both real and virtual conversations between service users, practitioners, policymakers and academics. In order to achieve this, we designed a series of interconnected work streams utilizing different data collection methods to facilitate discussion, as shown in Figure 1 below.

The research team consisted of survivor researchers and academics with practice experience. The principal investigator (SC) is a survivor researcher. Workstream 2 was led by survivor researchers (AF and SC with DG and CK), Work Streams 3 and 4 by practitioner academics (THL and CM, respectively) and Work Stream 5 by the team together. Two service user/survivor researchers were employed in Workstream 2.

Having shared aims and values and working to a set of agreed principles supported co‐productive working in the core team. This meant that collective approaches to decision making (including data analysis) were taken as far as was practicable within an institution that retains a hierarchical culture. The Project Advisory Group reflected the perspectives of the constituents to whom the researchers were accountable; it included service users, the police, mental health and adult safeguarding practitioners, researchers and policymakers. The end of project ‘sense‐making’ event attracted 42 people: social work practitioners, service users, carers, service providers, police, academics and policymakers explored the implications of study findings.

### 5.1 Ethical considerations

Ethical approval was obtained from Middlesex University Research Ethics Committee. We anticipated significant ethical issues because of the topic and the fact that the study population would be regarded as ‘vulnerable’. While research with 'vulnerable' adults has a number of ethical implications, affording mental health service users their right to a voice and to meaningful participation in research and practice is recognized as an ethical issue in itself.^26^ Managing this balance required the recognition that distress expressed in interviews when recalling traumatic or upsetting events is not necessarily equivalent to harm.^48^ People are often keen to continue if they feel safe and supported to do so.^26^ The research was conducted according to ethical principles of user‐led research, including key issues regarding transparency, respect, flexibility, accessibility, empowerment, a commitment to change, clarity about the underlying theoretical approach employed, accountability and to financially plan for participants' time and support needs.^26^, ^49^

The research conduct was informed by the SRA Code of Practice for the Safety of Social Researchers, which covers physical and emotional safety (http://the-sra.org.uk/wp-content/uploads/safety_code_of_practice.pdf), as well as the Ethics of Survivor Research.^26^

### 5.2 Control, consent and confidentiality

In keeping with our ethical principles, we aimed to give the research participants as much control over the process as we could, in order to minimize the power differential in the research relationship. In order to avoid any service provider or institutional obligation to participate, recruitment of participants took place through an open call via the National Survivor User Network (NSUN) across England, through social media and word of mouth, snowballing, and by direct visits to user groups and events by research team members.

Interview participants were assured of the confidentiality of the interview. The participant information sheet explained the circumstances in which confidentiality might need to be broken: disclosure about potential or actual harm to the individual or other persons, child protection issues or criminal disclosure. Confidentiality was maintained throughout the research including the report writing to ensure that no individual could be identified. Consent was seen as a continuous process with participants given a number of opportunities to withdraw. Participants were offered a copy of their interview transcript and given the choice to edit or withdraw it. Interviewees were given a shopping voucher and a 'thank you' card and received three email updates plus a copy of a summary of the study findings.

In practice, although some people did become distressed during interviews, no one chose to withdraw. A couple of participants took the opportunity of taking a break but expressed the wish to continue; a strong motivation for taking part in the research was the desire to prevent others experiencing what they had experienced, or at least to help people find ways through it.

I don’t perceive how change can occur if we don’t talk about what the problems are. […] someone has to do it and if that someone is me then I will do it. I don’t particularly like or enjoy going over some of these things, but I see no particular alternative and I want the world to be different from how it is now. People shouldn’t have to experience what I have.

### 5.3 Shared learning

For Workstream 2, we held two shared learning days in order to review our knowledge of qualitative interviewing and to address some of the key issues within the project. We each led on different aspects of this learning: CK led on sharing our personal knowledge of the topic; DG led on ethical issues relating to handling personal distress during interviewing; and AF and SC led on the planning and technical aspects of interviewing, approaching participants and arranging the interviews. This shared learning formed the basis of team building for the project.

### 5.4 Support and supervision for researchers

All researchers in the team had group and peer supervision, in the form of reflective research practice, and had opportunities for post‐interview debriefing. There was sufficient flexibility in the budget to allow for overnight stays, a meal with a supportive friend or accessible transport if the researcher needed them following a distressing interview. The research team reviewed the ethical conduct of the research on an ongoing basis, using survivor research ethical principles as guidance.^26^

### 5.5 Interview conduct

Interviews were semi‐structured, using a topic guide compiled by the service user researcher team, informed by the literature scoping review findings as well as full consultation with the Advisory Group. This explored:

- Service users' own narratives, concepts and experiences of mental health–related violence, abuse and hostility;
- Service users' own concepts of risk and staying safe;
- What service users do if they have been victimized; how and where do they access support; and is the support helpful;
- Advice and recommendations for others and for practice.

Although the topic guide provided a loose structure, the interviews were designed to be led by the participant and their narrative, with the aim of sharing control as far as possible—rather than replicating the dynamics and limitations that may have been experienced in previous service interventions, assessments or complaint procedures. We left it to the participants to choose their own examples and use their own definitions of abuse and risk. An open narrative approach enabled the participants’ accounts to lead the exploration of the issues. A post‐pilot ethical decision was made to allow the interviews to run for as long as the participant needed to tell their story and make sense of their experiences with the interviewer.

### 5.6 Analysis

The interviews were audio‐recorded and transcribed, and then subjected to a preliminary thematic analysis. These initial findings formed the basis for discussion in the practitioner and stakeholder focus groups and two Twitter discussions as well as to feed into the final 'sense‐making' event (see Figure 1). These subsequent stages represented the real and virtual conversations created by the methodology: the findings being taken to focus groups of social work practitioners and students, police, housing practitioners, service users and policymakers amongst others.^5^The initial analysis of the service user interviews drew on some of the principles of grounded theory^27^ such as transcript coding and the development of core categories and themes through comparative analysis.

The initial thematic analysis provided the basis for more detailed analysis using framework approach.^28^, ^29^ The service user researchers worked alongside academics with practice experience and the research assistant in order to share and co‐produce interpretations of the data.

### 5.7 Reflections on the process

We used two methods to enable us to learn from and about our research process. One was a short survey for interview participants to respond anonymously on their experiences of being involved in the study. The second was a reflective discussion between the team members, to ask ourselves and each other about the benefits and challenges of the methods we adopted.

## 6 KEY FINDINGS FROM THE MAIN STUDY

The study findings are reported in detail elsewhere.^1^ A summary of the key findings is given below:

- The majority of service users had histories of trauma and abuse and had experienced other forms of abuse such as racism and homophobia.
- Many did not report incidents because they did not feel they were ‘worth it’ and did not feel they would be believed because of their mental health or diagnosis.
- Living in fear of abuse and feeling unsafe were common across the service user interviews. Abusers, including some mental health staff, were thought to target victims in situations where individuals are vulnerable or powerless.
- Vulnerability, risk from others and feelings of powerlessness appeared to be determined by a person's individual situation, wider environment, diagnosis and/or relationships.
- The broader socio‐economic context of austerity could also increase the risks for some people where support from statutory (and voluntary and community) services is severely reduced.
- Neglect by mental health staff can be experienced as targeted abuse by service users, who also reported experiences of abuse, violence (including sexual) by staff as well as fellow service users in closed mental health service environments such as psychiatric wards and supported housing.
- Complicated, fragmented or absent responses from adult safeguarding, mental health and other services can result in further risk, distress and/or disengagement. This included a lack of support offered to people going through the safeguarding process.
- In responding to incidents, staff reported feeling disempowered, afraid to take responsibility, lacking in confidence to advocate for individuals or to ‘speak out’ about bad practice in such a system and in mental health or social work ‘blame cultures’.

### 6.1 Dissemination and impact strategies

A core principle of survivor research and co‐production is to bring about real change 26, 49; we designed a number of ways to maximize this possibility. Continuous knowledge exchange was integral to the research design and conduct. The data from the interviews were used in three ways: as research findings in their own right, for interpretation in the stakeholder and practitioner focus groups and for feeding into the Twitter‐facilitated discussion sessions to engage with a wider audience. The discussions engaged many service users as well as researchers and social work, police, housing and other safeguarding practitioners.

The study employed a combination of online, social media, conventional media and networked approaches to dissemination. The knowledge gained as a result of the research has been applied to specialist practitioner teaching at Middlesex University by THL working with DG and CK. The end of project ‘sense‐making’ event was designed to engage project partners and stakeholders in discussing the implications of the findings to see where the research could make the most impact. A variety of creative methods was employed to extend reach and promote impact. A graphic recording of the discussions during the day was made by a team from ‘More Than Minutes’, constituting another dissemination resource. We commissioned a 3‐minute animation of main themes by a service user arts enterprise, ‘Inkwell Arts and Media’ [https://www.inkwellarts.org.uk/portfolio-item/middlesex-university-london-hate-crime-animation/] which was shown at the event and has supported dissemination at conferences and online. One of the service user researchers (CK) produced an illustrated book of poetry based on her personal and experiential reflections about the study. The user‐led organization The NSUN will be commissioned to design an accessible evidence‐based resource for service users.

## 7 REFLECTIONS ON THE PROCESS

Although it is not possible to be certain about this, we have some evidence for the view that the approach we used resulted in different and richer data, revealing complexities and hitherto unconsidered aspects of risk, vulnerability, safety and trauma to be surfaced. Previous research indicates the value of reducing the distance between researcher and researched, for example involving service users as interviewers^50^, ^51^ and in the analysis and interpretation of results.^50^, ^52^, ^53^ Many share the view that service user interviewers can elicit more open and honest answers and obtain more in‐depth information, particularly where services themselves are under examination.^50^, ^54^, ^55^, ^56^, ^57^

In practice, the interviews revealed many examples of abuse, harm and neglect from within mental health and other services, including sexual abuse and assault, bullying and coercion. These findings support previous user‐led research,^18^, ^20^ where it is clear that staff and services themselves can represent a source of risk for people in distress. This can lead to a reluctance to speak openly about these experiences to people within the same system or service.

We adopted two methods for inviting reflections on the research process: one with participants and one between us as a research team.

### 7.1 Participants' experiences of the interviews

We invited participants to complete a brief semi‐structured questionnaire about their experience of taking part in the research. The survey was a combination of four Likert scale‐type questions, with a number of free‐text questions for people to respond in their own words. It was completed by nine of the 23 who took part. The questions were as follows:

1. How much influence did the fact that this research was led by people who've experienced mental health problems themselves (‘service user led’) have on your decision to take part?
2. How much difference did being able to talk to a researcher with experience of mental health problems make to you being able to talk openly in the interview?
3. How useful were the questions in helping you talk about your experiences?
4. How likely would you be to take part in service user led research again?

The results from this overall scoring and brief free‐text response thematic analysis are presented in Appendix A (Table 1). In summary, the results from this survey indicate that it made a positive difference to the participants that the research was led by people with experience of mental distress. All said it had encouraged them to take part and that it had enabled them to be more open in the interviews. Respondents used the open free‐text question in a number of ways, predominantly to support the project and user‐led research generally. Some thanked the individual researcher for their professionalism in conducting the interview (in one case citing being treated with ‘dignity and respect’), while others wished the research team well and said they hoped the research would make a difference. However, one respondent was pessimistic about impact, saying ‘I appreciate the purpose of your research…[but] I am sure nothing will come of it’.

Service users have better understanding of the issues faced by their peers

I just felt so much more comfortable and less alien

[The researchers]…are better placed to listen without prejudice

**Table 1:** Post‐interview survey results

*1. How much influence did the fact that this research was led by people who've experienced mental health problems themselves (‘service user led’) have on your decision to take part?*										
Not at all = 0		Slightly = 0			Somewhat = 0			Very = 5		Extremely = 4
Respondents cited flexibility, sensitivity, trust, understanding and research impact: ‘The researchers seemed far more flexible in their approach and were prepared to listen to issues…that were outside the original remit’. ‘Service users have better understanding of the issues faced by their peers’ ‘People with MH experience aren't just research for research sake’ ‘They recognise…the unique need of people they interview’ ‘Less likely to encounter offensive, condescending bullsh*t’										
*2. How much difference did being able to talk to a researcher with experience of mental health problems make to you being able to talk openly in the interview?*										
None = 0			Minor = 0			Moderate = 3			Major = 6	
Respondents cited empathy, comfort, confidence and positive experience of researcher ‘professionalism’: ‘I just felt so much more comfortable and less alien’ ‘I feel more confident and safe’ ‘[The researchers]…are better placed to listen without prejudice’ ‘Because those with experience of this kind of pain are more respectful of it’ ‘The researchers acted in a thoroughly engaging and professional manner…my confidence in them was as a result of this…’ ‘I am more confident that this is not just a tick‐box exercise’										
*3. How useful were the questions in helping you talk about your experiences?*										
Not at all = 1	Slightly = 0			Somewhat = 0			Moderately = 2			Extremely = 6
Respondents cited openness and flexibility during interviews, and the sensitivity given to asking questions, and the painfulness of talking about their experiences: ‘…the researchers allowed me to introduce topic I believe are very important rather than being too prescriptive about the questions…’ ‘They know what relevant questions to ask’ ‘It helped, but was of course also painful to talk about’										
*4. How likely would you be to take part in service user led research again?*										
Not at all = 0		Slightly = 0			Somewhat = 0			Very = 1		Extremely = 8
Respondents cited relevance of subjects, good experience of research process, being respected and valued, feeling safer with reduced fear of stigma and wanting their experience to make a difference: ‘…subjects are always relevant to us and not just in a clinical way but everyday life’ ‘I find peer‐led research respects participants, who feel more at ease sharing experiences they would not necessarily share with someone who doesn't have lived experience, for fear of stigma’ ‘I like to think that my experience might help others’ ‘I felt really listened to and perhaps of the most importance I was pleased that the researchers found a way of reaching people who are particularly hard to reach’ ‘They make you feel safe also value what you said and shared with you what they heard’ ‘I’m so encouraged by the process’										

### 7.2 Researcher reflections

The core research team (SC, THL, AF, DG, RC and CK) took part in two audio‐recorded sessions to reflect on the process and findings of the study. Some of these reflections are incorporated in the findings and recommendations to emerge from the research. However, it became clear that we shared some feelings that reflected the internalized invalidity of our participants: the sense that our research, like our participants' experiences, in revealing seriously painful experiences and difficult messages, might not be taken seriously or would be dismissed on the basis of being user‐led and 'biased'.

Then it's assumed, well, this is just because service users did this and, you know, they didn't use a proper research methodology and didn't analyse it properly. [quotation from research team discussion]

This became a powerful motivation for us to account for the research process in this paper: the desire to have our research and the voices of our participants taken seriously. We also reflected in our discussions that individual practitioners, organizations and society at large are insufficiently shocked or horrified by the sorts of incidents we heard about. We felt a strong sense of an institutional immunity to abuse reflected in practice, particularly on mental health inpatient wards.

Another feeling that became evident was the sense of helplessness that we felt as researchers listening to people's traumatic experiences. We could not do anything practical or ethical in real time to help people—only plan and hope for the best dissemination and impact that we could achieve. Nevertheless, this was a difficult feeling to experience.

I suppose I just felt this sense of alienation from the world for a while whilst I was going through these interviews. This kind of horrible sense of the world being a horrible place. [quotation from research team discussion]

We did stay in touch with a couple of the interviewees by email for a short while after the research. One asked for advice about contacting a lawyer to take up her case, and another just valued the opportunity to stay in touch. We discussed these incidents as a team and acted accordingly.

An unexpected outcome from involvement in the research was that one of the service user researchers was herself empowered by the knowledge she gained to engage with her own experiences of safeguarding.

I've been using the language that I've learned to articulate with practitioners and they are sitting up and listening when I'm speaking, because I use the language that, you know, needs no explanation. I'm using the right words. And I was actually asked 'well, what is it that you do?' and nobody has ever asked me that! [quotation from research team discussion]

## 8 CONCLUSIONS

For practice in this area to change, the experiential knowledge of service users exposed to victimization and abuse needs to be combined with practice and policy knowledge and research skills. We feel that this study effectively combined these knowledge and skills sets in a manner that privileged the experiential knowledge of participants and opened up spaces for dialogue with other stakeholders. The central purpose of emancipatory research 'is seen as supporting the empowerment of service users, securing their rights and needs and the making of broader social change… It is concerned primarily with improving people's lives rather than solely with generating knowledge’.^37^ ^p.17^ We cannot claim to have improved people's lives as a result of this research, but the findings offer evidence for commissioners and service providers to transform safeguarding practice, particularly in relation to mental health services.

## CONFLICT OF INTEREST

None.

## Data Availability

Data sharing is not applicable to this article as no new data were created or analysed in this study.

## Footnotes

1 Note: because of the study focus on adult safeguarding, we use the term ‘targeted violence and abuse’.

## Appendix A

### Keeping Control service user post‐interview Likert Scale and free‐text follow‐up question results

#### What were your expectations of the research?

Respondents hoped that the research would:

- have an impact on the quality of services, as well as stigma and discrimination that leads to the abuse of people with mental health problems;
- contribute new knowledge to mental health research in areas ‘not previously looked at and will be given more credence’;
- enable them as participants to share their experiences in order to make a difference to the experiences of others.

One respondent said they had ‘high’ expectations, while another said they had ‘no expectations’.

#### Is there anything else you would like to add?

Respondents used this open free‐text question to answer in a number of ways, predominantly to support the project and user‐led research generally. Some thanked the individual researcher for their professionalism in conducting the interview (in one case citing being treated with ‘dignity and respect’), while others wished the research team well and said they hoped the research would make a difference. However, one respondent was pessimistic about impact, saying ‘I appreciate the purpose of your research…[but] I am sure nothing will come of it’. One respondent acknowledged their role in ‘[helping] with the development of peer‐led research’, while another said it is ‘about time mental health service user are heard well done’.
